# Inhaled nitric oxide therapy and risk of renal dysfunction: a systematic review and meta-analysis of randomized trials

**DOI:** 10.1186/s13054-015-0880-2

**Published:** 2015-04-03

**Authors:** Sheng-Yuan Ruan, Tao-Min Huang, Hon-Yen Wu, Huey-Dong Wu, Chong-Jen Yu, Mei-Shu Lai

**Affiliations:** Institute of Epidemiology and Preventive Medicine, National Taiwan University, No.17 Xu-Zhou Road, Taipei, 10020 Taiwan; Division of Pulmonary and Critical Care Medicine, Department of Internal Medicine, National Taiwan University Hospital, Taipei, Taiwan; Department of Internal Medicine, Far Eastern Memorial Hospital, New Taipei City, Taiwan

## Abstract

**Introduction:**

Inhaled nitric oxide (iNO) is an important therapy for acute respiratory distress syndrome (ARDS), pulmonary hypertension and pediatric hypoxemic respiratory failure. Safety concerns regarding iNO and renal dysfunction have been reported; however, there are currently no systematic reviews on this issue. Our objective was to evaluate published randomized controlled trials (RCTs) to ascertain the risk of renal dysfunction associated with iNO therapy in patients with and without ARDS.

**Methods:**

A systematic review of databases was performed to identify RCTs which compared iNO with controls up to September 2014. Effect estimates for risk ratio (RR) of acute kidney injury (AKI) were pooled using a random-effects model.

**Results:**

Ten RCTs involving 1363 participants were included. Inhaled nitric oxide significantly increased the risk of AKI compared with controls (RR, 1.4, 95%CI, 1.06 to 1.83, p = 0.02). In the stratified analysis, a high cumulative-dose of iNO significantly increased the risk of AKI (RR, 1.52, 95%CI, 1.14 to 2.02, p = 0.004), whereas medium and low cumulative-doses did not (RR, 0.64, 95%CI, 0.23 to 1.81 and RR, 0.56, 95%CI, 0.11 to 2.86 respectively). In subgroup analysis by study population, an increased risk of AKI was observed in patients with ARDS (RR, 1.55, 95%CI, 1.15 to 2.09, p = 0.005) but not in those without (RR, 0.90, 95%CI, 0.49 to 1.67, p = 0.75).

**Conclusions:**

The available data show that iNO therapy may increase the risk of renal dysfunction, especially with prolonged use and in patients with ARDS. The risk in pediatric population is unknown owing to limited data. We suggest monitoring renal function during iNO therapy, and that future trials of iNO should evaluate renal safety.

**Electronic supplementary material:**

The online version of this article (doi:10.1186/s13054-015-0880-2) contains supplementary material, which is available to authorized users.

## Introduction

Nitric oxide was first reported to be an endogenous vasodilator in 1987 [1]. Soon after this discovery, inhaled nitric oxide (iNO) was applied to treat pulmonary hypertension and various pulmonary diseases [2,3]. Previous studies and clinical trials have shown that iNO possesses the therapeutic effects of selective pulmonary vasodilatation without causing systemic hypotension, and an effect on improving ventilation-perfusion mismatch [3,4]. Currently, iNO has been an important treatment modality for pulmonary hypertension, acute respiratory distress syndrome (ARDS), and pediatric hypoxemic respiratory failure [5-7].

Cumulative data from physiological studies and clinical trials suggest that iNO therapy has a good safety profile [8-10], and that its potential adverse effects, including methemoglobinemia, inhibition of platelet aggregation and systemic vasodilatation, are usually clinically insignificant [8,11,12]. However, a safety concern about renal dysfunction for nitric oxide inhalation was reported in a meta-analysis designed to evaluate the efficacy of iNO in ARDS [13]. This finding contradicts earlier evidence that iNO has favorable effects on renal and splanchnic perfusion [8]. In addition, this adverse effect, as a class effect of iNO therapy, was not observed in non-ARDS populations. These contrasting findings warrant further investigation to clarify the association between iNO and the risk of renal dysfunction in patients with and without ARDS. Therefore, we conducted this systematic review and meta-analysis to ascertain the risk of acute kidney injury (AKI) in iNO therapy and to investigate whether the risk varies among different patient populations.

## Materials and methods

### Search strategy

This systematic review was conducted using an a priori published protocol submitted to the PROSPERO website (Registration number CRD42013005731) and reported according to the Preferred Reporting Items for Systematic Reviews and Meta-Analyses (PRISMA) criteria [14]. No institutional review board approval or consents were needed for this systematic review because it evaluated published studies. We searched MEDLINE via the NCBI Entrez system and Cochrane Central Register of Controlled Trials up to 25 September 2014. Bibliographies of retrieved studies and recent review articles were also screened to identify additional trials. Both keywords and MeSH terms searches were used to identify relevant citations. The search terms were “inhaled nitric oxide” AND “randomized controlled trial”, and MeSH terms were “Nitric Oxide/therapeutic use” [Mesh] AND “Randomized Controlled Trial” [Publication Type]. There were no language restrictions. Details of our search strategy are shown in the online appendix (see Additional file 1). The eligible studies were parallel randomized controlled trials (RCTs) comparing patients who did and did not receive iNO therapy. The exclusion criteria included crossover trials and studies not reporting AKI in the results.

### Study selection and data extraction

Two investigators (SYR and TMH) screened studies for inclusion and independently extracted data from the included studies. Any disagreements were resolved by discussion. A standardized recording form was used for data extraction.

### Assessment of risk of bias

Quality assessment of these studies was done using the Cochrane Collaboration tool for assessing the risk of bias [15]. We assessed the risk of bias associated with random sequence generation, allocation concealment, blinding of participants and personnel, blinding of outcome assessments, incomplete outcome data, selective outcome reporting, and other bias. Two investigators (SYR and TMH) independently assessed the risk of bias in the individual studies, and discrepancies were resolved by consensus.

### Outcome measures

The primary outcome of interest was AKI as defined in the individual studies regardless of the severity of injury. The secondary outcome was AKI requiring renal replacement therapy (RRT). Risk ratio (RR) was used as a measurement of association in the analysis.

### Stratified analysis

Stratified analysis by study population was performed to investigate whether the risk of iNO-associated renal dysfunction varied among different patient populations. To test the dose-response relationship between iNO and the risk of renal dysfunction, stratified analysis by duration and dosage of iNO therapy was also performed.

### Data synthesis

Outcome measures were pooled using a random-effects model with the inverse variance method because of anticipated heterogeneity among the included studies. We analyzed data by intention-to-treat analysis and calculated the point estimates and 95% CI of the summary effect. When one arm of a study contained no events, 0.5 was added to all cells of the two-by-two table [16]. Sensitivity analysis was performed to check the robustness of the effect estimate using different data synthesis models. The Peto method was used in the sensitivity analysis because of its advantage of a less biased estimate for summary effect in the settings of sparse data and imbalance of trial size [17]. Because the Peto method can only be used to calculate odds ratios (ORs), we also calculated ORs using a random-effects model to compare the effect sizes obtained by these two methods. Heterogeneity was explored using the *Q*-statistic and I^2^. Heterogeneity was considered low, moderate, and high according to I^2^ values of <25%, 25 to 50% and >50%, respectively [18]. Publication bias was evaluated by a funnel plot of standard error versus RR based on the primary outcome, and with Egger’s test [19]. For all analyses, a two-sided *P*-value <0.05 was considered to be statistically significant. The data were analyzed using Stata software, version 11 (StataCorp, Texas, USA).

## Results

### Literature search and study characteristics

**Figure 1:**
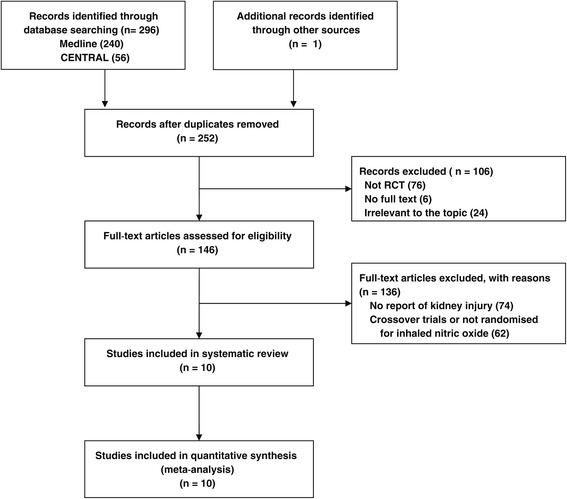
**Study flow through this systematic review.** RCT, randomized controlled trial.

Through the electronic searches and from references, 252 citations were identified. According to our predefined inclusion and exclusion criteria, 10 RCTs involving a total of 1,363 patients were included in the final analysis [12,20-28]. To calculate the risk for incident AKI, 26 patients were excluded from the analysis because they had received hemodialysis before the enrollment of trials. The number of studies evaluated at each stage of the literature review is shown in Figure 1. Quality assessment (see Additional file 2) of the included studies suggested a low risk of bias, except for one study which was published with an abstract [21].

The characteristics of the included studies are summarized in Table 1. The publication year ranged from 1998 to 2014, and included four studies on patients with ARDS, four studies on patients undergoing surgery, one study on neonates with hypoxemic respiratory failure, and one study on patients with sepsis. The administered dose and duration of iNO varied largely among these studies. In the ARDS trials, the dose of iNO shifted from a titrated dose of 1 to 40 ppm in the 1990s to a fixed dose of 5 ppm in the latest trial [12,23]. The treatment duration was longer in the ARDS studies (>7 days) than in the non-ARDS studies (≤7 days).

**Table 1:** **Details of the included randomized controlled trials**

**Study (year)**	**Country**	**Study population**	**Protocol of iNO therapy**	**Mean iNO dosage**	**Age, years**	**Definition of acute kidney injury (AKI)**	**Number of AKI/number of cases**	
							**iNO**	**Control**
Dellinger (1998) [12]	USA	ARDS	1.25, 5.0, 20.0, 40.0, or 80.0 ppm iNO for 28 days or till FiO_2_ < 0.5	21 ppm	48	Creatinine >2 mg/dL	20/120	7/57
						Creatinine ≥3.5 mg/dL	13/120	5/57
Lundin (1999) [20]	11 European countries	ARDS	1 to 40 ppm iNO at the lowest effective dose for up to 30 days or until an end point was reached	9 ppm	57	Creatinine >3.4 mg/dL or RRT	28/80	12/74
						Incident RRT	23/84	10/79
Kinsella (1999) [22]	USA	Neonate hypoxemic respiratory failure	5 ppm for 7 days	5 ppm	27 weeks	Renal failure	2/48	2/32
Payen (1999) [21]	Europe	ARDS	10 ppm till PF >250, median 5 days	10 ppm	Not reported	RRT	33/98	26/105
Taylor (2004) [23]	USA	ARDS	5 ppm until 28 days, discontinuation of assisted breathing, or death	5 ppm	50	Creatinine ≥3 mg/dL	12/192	8/193
						Creatinine ≥3.5 mg/dL	10/192	6/193
Perrin (2006) [24]	France	Lung transplantation	20 ppm for 12 h	20 ppm	35	RRT	1/15	1/15
Potapov (2011) [26]	USA and Germany	Cardiac surgery	40 ppm for 48 h	40 ppm	56	RRT	10/73	8/77
Fernandes (2011) [25]	Brazil	Cardiac surgery	10 ppm for 48 h	10 ppm	46	Urine output <0.3 ml/kg/h	0/14	1/15
Lang (2014) [28]	USA	Liver transplantation	80 ppm during the operative phase	80 ppm	56	Renal dysfunction	3/40	7/40
Trzeciak (2014) [27]	USA	Sepsis	40 ppm for 6 h	40 ppm	59	RRT	2/26	1/23

AKI, acute kidney injury; ARDS, acute respiratory distress syndrome; iNO, inhaled nitric oxide; FiO_2_: inspired oxygen fraction; PF: PaO_2_/FiO_2_ ratio; RRT: renal replacement therapy.

### Reporting of renal dysfunction

Renal dysfunction in these trials was usually defined as an excess of creatinine level to a predefined level or the need for renal replacement therapy (Table 1). Among these trials, three reported the data of two different definitions of renal dysfunction. Notably, many clinical trials of iNO were excluded from this systematic review due to a lack of data on renal adverse effects, especially in the pediatric studies. Bleeding and neurological complications were the main concerns for the pediatric patients, and data on renal dysfunction were rarely reported in the safety outcomes.

### Quantitative data synthesis

**Figure 2:**
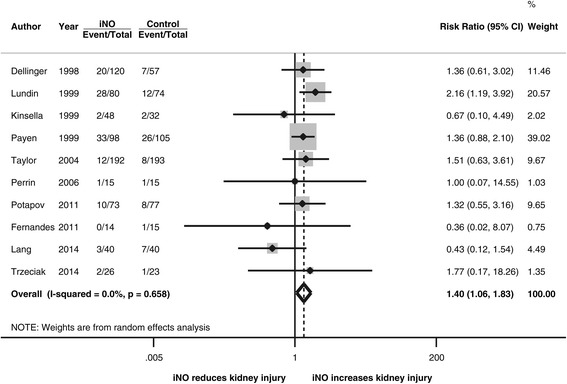
**Forest plot for the risk of acute kidney injury.** iNO, inhaled nitric oxide.

For the primary outcome of AKI with any severity (Figure 2), the pooled effect from 10 studies showed that iNO therapy significantly increased the risk of AKI with an RR of 1.40 (95% CI, 1.06 to 1.83, *P* = 0.02, *I*^2^ = 0%). The study by Payen [21] accounted for 39% of meta-analysis weight but was only published with an abstract. After omitting this influential study, the effect estimate remained similar (RR, 1.42, 95% CI, 1.003 to 2.01, *P* = 0.048, *I*^2^ = 0%). The statistical heterogeneity was low among the analyses.

**Figure 3:**
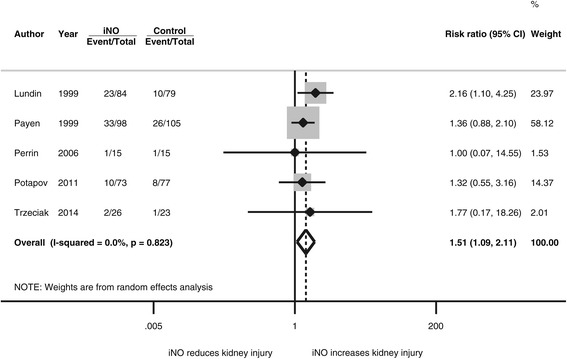
**Forest plot for the risk of initiating renal replacement therapy.** iNO, inhaled nitric oxide.

For the endpoint of AKI requiring renal replacement therapy (Figure 3), iNO also increased the risk with a RR of 1.51 (95% CI, 1.09 to 2.11, *P* = 0.01, *I*^2^ = 0%). The effect estimate became larger after we omitted the influential study by Payen (RR, 1.76, 95% CI, 1.05 to 2.93, *P* = 0.03, *I*^2^ = 0%).

Due to the presence of sparse data and imbalance of trial size, sensitivity analysis was performed to evaluate the influence of data synthesis methods on the estimate of summary effect. The effect estimate by the Peto method was similar to that obtained by the primary analysis with a random-effects model (Table 2).

**Table 2:** **Sensitivity analysis by different data synthesis methods**

**Outcome measures**	**Number of studies (number of patients)**	**Statistical model**	**Effect size (95% CI)**	***P*** **-value (test for effect)**	**Heterogeneity**
Acute kidney injury	10 (1337)	RR, random-effects	1.40 (1.06 to 1.83)	0.02	*I* ^2^ = 0%
		OR, random-effects	1.50 (1.07 to 2.09)	0.02	*I* ^2^ = 0%
		OR, Peto	1.48 (1.07 to 2.05)	0.02	*I* ^2^ = 0%
Initiation of renal replacement therapy	5 (595)	RR, random-effects	1.51 (1.09 to 2.11)	0.01	*I* ^2^ = 0%
		OR, random-effects	1.73 (1.13 to 2.65)	0.01	*I* ^2^ = 0%
		OR, Peto	1.73 (1.14 to 2.41)	0.01	*I* ^2^ = 0%

RR, risk ratio; OR, odds ratio.

**Figure 4:**
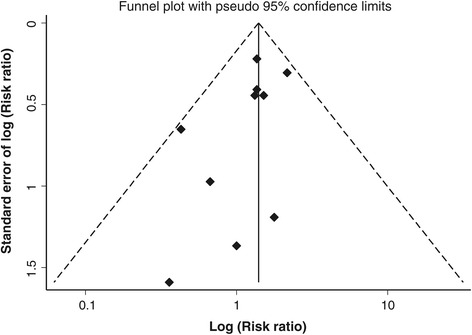
**Funnel plot based on the primary outcome.**

Figure 4 shows a funnel plot based on the primary outcome. The asymmetry of the funnel plot on visual inspection implied a lack of studies in which iNO increased the risk of AKI. This suggests that the pooled effect from the current data may underestimate the effect size of the risk. However, the statistical test for publication bias, Egger’s test (*P* = 0.33), did not reach statistical significance.

The risk of AKI associated with iNO therapy varied among the different populations (Table 3). The risk was significantly increased in the patients with ARDS (RR, 1.55, 95% CI, 1.15 to 2.09, *P* = 0.005), but not in patients without ARDS (RR, 0.9, 95%CI, 0.49 to 1.67, *P* = 0.75). Among the patients with ARDS, the risk difference for AKI between iNO and control groups was 0.067 (95% CI, 0.000 to 0.135, *P* = 0.05, *I*^2^ = 50%), and the number needed-to-harm to cause one additional AKI was 15.

**Table 3:** **Subgroup analysis by study population**

**Subgroups**	**Number of studies (number of patients)**	**Risk ratio of AKI (95% CI)**	***P*** **-value (test for effect)**	**Heterogeneity**
ARDS	4 (919)	1.55 (1.15 to 2.09)	0.005	*I* ^2^ = 0%
Non-ARDS	6 (418)	0.90 (0.49 to 1.67)	0.75	*I* ^2^ = 0%
Surgery	4 (289)	0.89 (0.45 to 1.75)	0.73	*I* ^2^ = 0%
Sepsis	1 (49)	1.77 (0.17 to 18.26)	0.63	Not applicable
Pediatric hypoxemic respiratory failure	1 (80)	0.67 (0.10 to 4.49)	0.68	Not applicable

AKI, acute kidney injury; ARDS, acute respiratory distress syndrome.

**Figure 5:**
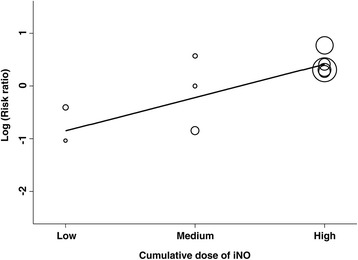
**Bubble plot with fitted meta-regression line depicting the relationship between the risk of renal dysfunction and cumulative dose of inhaled nitric oxide (Ino).**

To test the dose-response relationship between iNO and the risk of AKI, we performed stratified analysis by duration and dosage of iNO therapy. Prolonged use of iNO (>7 days) significantly increased the risk of AKI (RR, 1.55, 95% CI, 1.15 to 2.09, *P* = 0.005, four studies), whereas short-term use did not (RR, 0.90, 95% CI, 0.49 to 1.67, *P* = 0.75, six studies). Notably, the four studies involving the prolonged use of iNO are all ARDS studies. Table 4 summarizes the risk of renal dysfunction for different iNO exposure levels. We classified the included studies into three groups according to the cumulative dose in the stratified analysis. High cumulative dose of iNO significantly increased the risk of renal dysfunction but medium and low cumulative doses did not (Table 4). Figure 5 depicts the relationship between the risk of renal dysfunction and cumulative dose of iNO. Visual inspection suggested a possible association between the cumulative dose and risk of renal dysfunction but statistical test by meta-regression analysis was not significant due to small sample size (*P* = 0.10).

**Table 4:** **Dose-response relationship between inhaled nitric oxide and the risk of acute kidney injury**

**Cumulative dose of inhaled nitric oxide**	**Numer of studies (number of patients)**	**Risk ratio of AKI (95% CI)**	***P*** **-value (test for effect)**	**Heterogeneity**	**References**
Low	2 (109)	0.56 (0.11 to 2.86)	0.49	*I* ^2^ = 0%	[22,25]
Medium	3 (159)	0.64 (0.23 to 1.81)	0.40	*I* ^2^ = 0%	[24,27,28]
High	5 (1069)	1.52 (1.14 to 2.02)	0.004	*I* ^2^ = 0%	[12,20,21,23,26]

AKI, acute kidney injury.

## Discussion

This meta-analysis updated the evidence on the renal safety of iNO therapy in ARDS and non-ARDS populations. The study suggested that the risk of iNO-associated renal dysfunction differed between ARDS and non-ARDS populations. The results showed that iNO therapy was associated with a 40% increased risk of renal dysfunction and the observed effect was mainly attributed to ARDS studies with prolonged use of iNO. If treating ARDS patients with iNO the number needed-to-harm to cause one additional AKI was 15. Sensitivity analysis by different data pooling methods revealed consistent effect estimates, and publication bias analysis suggested that the risk may be underestimated. Clinicians should be aware of this side effect when using iNO to treat patients. Our review also found that the safety outcome of renal dysfunction was not universally reported across iNO trials, especially in non-ARDS studies. We suggest that future trials of iNO should report renal safety outcomes.

The risk of renal dysfunction associated with iNO therapy was first reported in an RCT of patients with ARDS, in which iNO was found to double the risk of the need for renal replacement therapy compared with controls [20]. However, this adverse effect did not raise much attention because subsequent trials of iNO reported no significant increase in the risk of renal dysfunction [12,23]. In 2007, Adhikari and colleagues reported that iNO was associated with renal dysfunction in a meta-analysis [13]. This meta-analysis only included ARDS trials because the primary study interest was the efficacy of iNO on ARDS [13]. The strengths of our study are the comprehensive evaluations of iNO-related renal dysfunction among patients with and without ARDS, the influence of different endpoints of renal dysfunction, and the dose-response relationship. Although our analysis suggests that iNO may increase the risk of renal dysfunction, it is not clear whether the increased risk is ARDS-specific or related to prolonged use of iNO. We were unable to clarify this in study-level analysis because the ARDS studies all involved the prolonged use of iNO.

Generating awareness of the nephrotoxicity of iNO has important implications for preventing AKI in the intensive care unit. Previous studies have shown that the development of AKI significantly increased mortality in patients with ARDS [29]. We suggest that intensivists should be cautious about the concurrent use of iNO with other nephrotoxic agents such as certain antibiotics, when treating ARDS. Renal function should be regularly monitored during iNO therapy to detect possible AKI early. In addition, the nephrotoxicity of iNO may partly explain why iNO therapy improves oxygenation and attenuates inflammation but confers no benefit on mortality in patients with ARDS [3,30,31]. The benefits of iNO may be counterbalanced by the increased risk of renal failure, because organ failure is positively associated with mortality [32].

The mechanism of iNO-related renal dysfunction remains unclear. Table 5 summarizes the findings of animal and physiological studies on this issue [9,33-38]. These studies all examined the effect of short-term exposure to iNO, and most reported that iNO had beneficial effects on renal function except for a swine study which reported that iNO promoted renal tubular apoptosis [33]. In this swine model, renal resorption capacity was significantly blunted by iNO, and this may have led to tubular and glomerular injury by means of tubuloglomerular feedback [33]. With respect to nitric oxide metabolites, nitric oxide inhalation increased serum levels of plasma cyclic guanosine monophosphate (cGMP), nitrate, and nitrite [39]. These nitric oxide metabolites may play a role in AKI development owing to the effects on protein nitrosation and raising oxidative load [40-42]. In an animal model of drug-induced nephrotoxicity, elevated nitrite and nitrate levels were observed with increasing oxidative activity [40]. The induced nephrotoxicity could be ameliorated by selective phosphodiesterase-5 inhibitor by suppressing oxidative activity and nitrite and nitrate levels [40]. In addition, from the pathways of chemical reactions of nitric oxide in the lung, we speculate that oxidative injury caused by highly oxidant nitrogen compounds may also play a role in iNO-related kidney injury. Reactive nitrogen species such as nitrogen dioxide (NO_2_) are highly oxidative compounds that are produced when iNO mixes with high concentrations of oxygen in the alveoli [3,11]. In patients with ARDS treated with iNO, the serum concentrations of NO_2_ have been positively associated with the dose of iNO administered [12]. It has also been shown that systemically circulating NO_2_ may lead to cytotoxic effects on renal parenchymal cells [43]. This hypothesis of oxidative injury by reactive nitrogen species may also explain why nephrotoxicity tends to develop in patients with ARDS receiving iNO therapy, as these patients usually receive high-concentration oxygen therapy, which may facilitate the production of reactive nitrogen species. Animal studies are needed to test this hypothesis.

**Table 5:** **Animal and human studies investigating the effects of inhaled nitric oxide (iNO) on the kidneys**

**Study (year)**	**Species**	**Protocols of iNO**	**Main findings**
Valvini (1995) [38]	Human	40 ppm for 3 days followed by 90 ppm for 2 days	1. Inhaling 40 ppm nitric oxide would result in a daily nitrogen oxide load of about 25 mmol.
			2. Impairment of renal function would cause an increase in serum nitrogen oxides.
Troncy (1997) [9]	Swine	40 ppm iNO	Inhaled nitric oxide increased renal blood flow, glomerular filtration rate and urinary flow.
Preiser (1998) [37]	Human	1 to 20 ppm	1. Renal excretion of NO_2_^−^ and NO_3_^−^ was unaltered by nitric oxide inhalation.
			2. Long-term nitric oxide inhalation was associated with a consistent increase in the NO_3_^−^ plasma concentration.
Wraight (2001) [36]	Human	40 ppm for 2 h	Inhaled nitric oxide may alter tubular salt and water resorbtion.
Kielbasa (2001) [35]	Rat	49 or 107 ppm iNO for 4 h	High dose of iNO increased nitric oxide synthase III protein expression, and nitrotyrosine and phosphotyrosine immunoreactivity.
Da (2007) [34]	Swine	30 ppm iNO for 3.5 h	Decreased swelling and necrosis of glomeruli.
Gozdzik (2009) [33]	Swine	40 ppm iNO for 30 h	1. Transient natriuretic effect.
			2. Renal tubular apoptosis promotion after 30 h of iNO treatment.
Göranson (2014) [44]	Swine	30 ppm iNO for 30 h	Combined therapy with iNO and intravenous steroid is associated with partial protection of kidney function.

### Limitations

Our study has limitations. First, the included number of studies and the number of cases in individual studies are small for the non-ARDS trials. This may have limited the statistical power of our subgroup analysis and meta-regression. Second, although the level of statistical heterogeneity was very low in all of our analyses, the heterogeneity on exposure (treatment duration and dosage of iNO) should not be overlooked. Third, death was a competing risk factor in our analysis. However, we were unable to adjust for this competing risk in study-level analysis. Individual data are needed to conduct such analyses.

## Conclusion

This meta-analysis updated the evidence on the renal safety of iNO therapy in ARDS and non-ARDS populations. Our results suggest that iNO therapy substantially increases the risk of renal dysfunction. It remains unclear whether this markedly increased risk is disease-specific in ARDS or related to prolonged use of iNO. Further studies are needed to clarify this issue. We suggest that renal function should be monitored during iNO therapy and that future clinical trials of iNO should evaluate renal safety.

## Key messages

- Previous studies have shown that iNO has a good safety profile and favorable effects on renal and splanchnic perfusion; however, iNO therapy has been reported to be associated with renal dysfunction.
- This meta-analysis updated the evidence regarding renal safety of iNO therapy. The study suggested that the risk of iNO-associated renal dysfunction differed between ARDS and non-ARDS populations. Nitric oxide inhalation may increase the risk of renal dysfunction, especially with prolonged use and in patients with ARDS.
- The safety outcome of renal dysfunction was not universally reported across iNO trials, especially in non-ARDS studies. We suggest monitoring renal function during iNO therapy, and that future trials of iNO should evaluate renal safety.

## Additional files

Additional file 1:
**Details of the search strategy and results.**
Additional file 2:
**Quality assessment using the Cochrane risk of bias tool.**

## Abbreviations

AKI: acute kidney injury
ARDS: acute respiratory distress syndrome
iNO: inhaled nitric oxide
OR: odds ratio
PRISMA: Preferred Reporting Items for Systematic Reviews and Meta-Analyses
RCT: randomized controlled trial
RR: risk ratio
RRT: renal replacement therapy
