# Supplementary material for: Inhaled nitric oxide therapy and risk of renal dysfunction: a systematic review and meta-analysis of randomized trials
Source: Crit Care. 2015 Apr 3;19(1):137. doi: 10.1186/s13054-015-0880-2 (PMC4384233; doi:10.1186/s13054-015-0880-2)
Supplement: Additional file 1: — Details of the search strategy and results. [file 13054_2015_880_MOESM1_ESM.pdf]

Table S1. Details of the search strategy and results (Search date: September 25, 2014)

Step	Database and search items	No. of citations
	**MEDLINE via the NCBI Entrez system**	
#1	"inhaled nitric oxide" AND "randomized controlled trial"	195
#2	"Nitric Oxide/therapeutic use"[Mesh] AND "Randomized Controlled Trial" [Publication Type]	211
#3	#1 OR #2	240
	**Cochrane Central Register of Controlled Trials**	
#4	"inhaled nitric oxide" AND "randomized controlled trial"	56
