# Supplementary material for: Inhaled nitric oxide therapy and risk of renal dysfunction: a systematic review and meta-analysis of randomized trials
Source: Crit Care. 2015 Apr 3;19(1):137. doi: 10.1186/s13054-015-0880-2 (PMC4384233; doi:10.1186/s13054-015-0880-2)
Supplement: Additional file 2: — Quality assessment using the Cochrane risk of bias tool. [file 13054_2015_880_MOESM2_ESM.pdf]

Table S2. Quality assessment using the Cochrane risk of bias tool

Study	Random sequence generation	Allocation concealment	Blinding of participants and researchers	Blinding of outcome assessment	Incomplete outcome data	Selective reporting	Other bias
Dellinger (1998)[1]	Low risk	Low risk	Low risk	Low risk	Low risk	Low risk	Low risk
Lundin (1999)[2]	Low risk	Unclear risk	Unclear risk	Low risk	Low risk	Low risk	Low risk
Kinsella (1999)[3]	Low risk	Low risk	Low risk	Low risk	Low risk	Low risk	Low risk
Payen (1999)[4]	Unclear risk	Unclear risk	Unclear risk	Unclear risk	Unclear risk	Unclear risk	Unclear risk
Taylor (2004)[5]	Low risk	Low risk	Low risk	Low risk	Low risk	Low risk	Low risk
Perrin (2006)[6]	Unclear risk	Unclear risk	Unclear risk	Low risk	Low risk	Low risk	Low risk
Potapov (2011)[7]	Low risk	Low risk	Low risk	Low risk	Unclear risk	Low risk	Low risk
Fernandes (2011)[8]	Unclear risk	Low risk	Low risk	Low risk	Low risk	Low risk	Low risk
Lang (2014)[9]	Low risk	Low risk	Low risk	Low risk	Unclear risk	Low risk	Low risk
Trzeciak (2014)[10]	Low risk	Low risk	Low risk	Low risk	Low risk	Low risk	Low risk

References

1. Dellinger RP, Zimmerman JL, Taylor RW, Straube RC, Hauser DL, Criner GJ, Davis K, Jr., Hyers TM, Papadakos P: **Effects of inhaled nitric oxide in patients with acute respiratory distress syndrome: results of a randomized phase II trial. Inhaled Nitric Oxide in ARDS Study Group**. *Crit Care Med* 1998, **26**:15-23.
2. Lundin S, Mang H, Smithies M, Stenqvist O, Frostell C: **Inhalation of nitric oxide in acute lung injury: results of a European multicentre study. The European Study Group of Inhaled Nitric Oxide**. *Intensive Care Med* 1999, **25**:911-919.
3. Kinsella JP, Walsh WF, Bose CL, Gerstmann DR, Labella JJ, Sardesai S, Walsh-Sukys MC, McCaffrey MJ, Cornfield DN, Bhutani VK *et al*: **Inhaled nitric oxide in premature neonates with severe hypoxaemic respiratory failure: a randomised controlled trial**. *Lancet* 1999, **354**:1061-1065.
4. Payen D, Vallet B, Group d'étude du NO dans l'ARDS: **Results of the French prospective multicentric randomized double-blind placebo-controlled trial on inhaled nitric oxide (NO) in ARDS [Abstract]**. *Intensive Care Med* 1999, **25**:166.
5. Taylor RW, Zimmerman JL, Dellinger RP, Straube RC, Criner GJ, Davis K, Jr., Kelly KM, Smith TC, Small RJ: **Low-dose inhaled nitric oxide in patients with acute lung injury: a randomized controlled trial**. *JAMA* 2004, **291**:1603-1609.
6. Perrin G, Roch A, Michelet P, Reynaud-Gaubert M, Thomas P, Doddoli C, Auffray JP: **Inhaled nitric oxide does not prevent pulmonary edema after lung transplantation measured by lung water content: a randomized clinical study**. *Chest* 2006, **129**:1024-1030.
7. Potapov E, Meyer D, Swaminathan M, Ramsay M, El Banayosy A, Diehl C, Veynovich B, Gregoric ID, Kukucka M, Gromann TW *et al*: **Inhaled nitric oxide after left ventricular assist device implantation: a prospective, randomized, double-blind, multicenter, placebo-controlled trial**. *J Heart Lung Transplant* 2011, **30**:870-878.
8. Fernandes JL, Sampaio RO, Brandao CM, Accorsi TA, Cardoso LF, Spina GS, Tarasoutchi F, Pomerantzeff P, Auler JO, Jr., Grinberg M: **Comparison of inhaled nitric oxide versus oxygen on hemodynamics in patients with mitral stenosis and severe pulmonary hypertension after mitral valve surgery**. *Am J Cardiol* 2011, **107**:1040-1045.
9. Lang JD, Jr., Smith AB, Brandon A, Bradley KM, Liu Y, Li W, Crowe DR, Jhala NC, Cross RC, Frenette L *et al*: **A randomized clinical trial testing the anti-inflammatory effects of preemptive inhaled nitric oxide in human liver transplantation**. *PLoS One* 2014, **9**:e86053.

10. Trzeciak S, Glaspey LJ, Dellinger RP, Durflinger P, Anderson K, Dezfulian C, Roberts BW, Chansky ME, Parrillo JE, Hollenberg SM: **Randomized Controlled Trial of Inhaled Nitric Oxide for the Treatment of Microcirculatory Dysfunction in Patients With Sepsis**. *Crit Care Med* 2014, **42**:2482-2492.
